# Dating the Diversification of the Major Lineages of Ascomycota (Fungi)

**DOI:** 10.1371/journal.pone.0065576

**Published:** 2013-06-14

**Authors:** María Prieto, Mats Wedin

**Affiliations:** 1 Department of Botany, Swedish Museum of Natural History, Stockholm, Sweden; 2 Departamento de Biología y Geología, Universidad Rey Juan Carlos, Móstoles, Madrid, Spain; University of Sydney, Australia

## Abstract

Establishing the dates for the origin and main diversification events in the phylogeny of Ascomycota is among the most crucial remaining goals in understanding the evolution of Fungi. There have been several analyses of divergence times in the fungal tree of life in the last two decades, but most have yielded contrasting results for the origin of the major lineages. Moreover, very few studies have provided temporal estimates for a large set of clades within Ascomycota. We performed molecular dating to estimate the divergence times of most of the major groups of Ascomycota. To account for paleontological uncertainty, we included alternative fossil constraints as different scenarios to enable a discussion of the effect of selection of fossils. We used data from 6 molecular markers and 121 extant taxa within Ascomycota. Our various ‘relaxed clock’ scenarios suggest that the origin and diversification of the Pezizomycotina occurred in the Cambrian. The main lineages of lichen–forming Ascomycota originated at least as early as the Carboniferous, with successive radiations in the Jurassic and Cretaceous generating the diversity of the main modern groups. Our study provides new information about the timing of the main diversification events in Ascomycota, including estimates for classes, orders and families of both lichenized and non–lichenized Ascomycota, many of which had not been previously dated.

## Introduction

Molecular dating, or the use of DNA sequences to estimate divergence times in phylogenetic trees, is rapidly developing into one of the most exciting applications of phylogenetic systematics [1]. Common tools for estimating evolutionary timescales are often based on the molecular clock hypothesis, which assumes a constant evolutionary rate over time [2]. Unsurprisingly, however, deviations from a clocklike evolution are found in many cases [3]–[6]. Different methods have been developed to handle rate heterogeneity [7], some of which attempt to relax the molecular clock assumption by allowing the rate to vary across the tree [8]–[11]. These relaxed–clock methods offer greater flexibility in modelling evolutionary events and in incorporating calibrations, which has resulted in a recent discussion about how to calibrate divergence time estimates [11]–[16]. Calibrations play a crucial role in studies of divergence times [12], [16], [17] and incorrect calibrations can introduce error into an analysis [18]. Sources of error in the calibration process include the incompleteness of the fossil record, erroneous fossil age estimates, and erroneous placements of fossils on phylogenetic trees [19]. Different methods deal with uncertainty associated with the phylogenetic position of fossil calibration points [20]–[22]. The method implemented in the Bayesian phylogenetic software BEAST [23] allows the user to account for uncertainty in the age of a given fossil, in the form of a prior distribution. By allowing calibrating information to be represented in the form of parametric distributions, this approach offers a high degree of flexibility to incorporate a time scale into a phylogenetic analysis [16].

Several estimates for divergence times in Fungi have been published during the last two decades (e.g. [24]–[27]). Many of these studies obtained radically different age estimates for the same divergence events, with analyses based on different methods (e.g. single rate, strict clock), datasets (based on a single gene, or more), taxon sampling, and fossil constraints, including external divergences (e.g. the split between eudicots and monocots, or extrapolations from animal divergences) or secondary calibrations (i.e. a point derived from a previous study). The fossil record in Fungi is very limited compared with plants and animals [28]. This is due to a combination of the often microscopic nature of fungi, the very poor preservation potential [29] and probably, the relative difficulty in recognizing them in the fossil record [30]. Many, probably most, major lineages of fungi thus currently lack fossil data.

As the fossil record is fragmentary, a given fossil will very rarely possess features that place it in the crown group rather than along the stem lineage leading to the crown group [19]. Donoghue & Purnell [31] point out that the most conservative and secure interpretation of the fossils is to avoid trying to resolve equivocal interpretations of stem or crown group classifications and instead to accept their classification as part of the total group. In Fungi, one of the most noteworthy examples is *Paleopyrenomycites devonicus*, the oldest unequivocal euascomycete fossil from the lower Devonian (ca 400 million years ago [Mya]), the systematic position of which has been widely disputed [26]–[27], [32]–[34]. Lücking et al. [32] placed this fossil at three different positions in the Ascomycota (i.e. at the origin of Sordariomycetes, at the divergence of Pezizomycotina, and at the origin of Pezizomycotina) and re–estimated the divergence times for main fungal groups. This was done through a graphical recalibration of nodes from a number of fungal molecular clock trees, by fixing the age of the Ascomycota-Basidiomycota origin to 1 and calculating the relative ages of major nodes in the tree ([32], pp 814–815). The fossil is morphologically complex and does not fit within any extant taxonomic group. The presence of an ascoma opening similar to an operculum has resulted in different interpretations about its affinities, although it is clear that this structure is not homologous with the operculum in modern Pezizomycotina. Consequently, a conservative approach is to treat this fossil as a member of the stem lineage of the Ascomycota subphylum Pezizomycotina (the “Euascomycetes”).

Other remarkable Ascomycota fossils found in amber include an *Aspergillus* species growing on a springtail from the Eocene [35], a species related to the extant anamorphic ascomycete genus *Curvularia* from the Cretaceous [36] and a *Xylaria* species from Dominican amber [37]. The resinicolous mazaediate fungus *Chaenothecopsis bitterfeldensis* was found in Bitterfeld amber from around 22 million years ago, near the Miocene–Oligocene boundary [38] and recently, two *Chaenothecopsis* fossils were described from Eocene Baltic and Oligocene Bitterfeld amber dating back to at least 35 and 24 Ma ago, respectively [39].

Well–preserved lichen fossils are found in Baltic (35–55 Mya) and Dominican amber (with estimates ranging from 15–20 to 30–45 Mya). Among these fossils, there is an alectorioid lichen and two specimens of *Anzia* from Baltic amber [40]–[41], two species of *Parmelia* s.l. and one *Phyllopsora* described from Dominican amber [42]–[43]. Crustose lichen fossils belonging to both mazaediate genera *Calicium* and *Chaenotheca* were also found in Baltic amber [44]. Finally, an impression of a foliose macrolichen belonging to Lobariaceae [45] was found from Miocene deposits.

Until now, very few studies have included these fossils to calibrate phylogenetic trees and even fewer have used them to estimate the timing of the main divergence events within Ascomycota. Most of the existing dating studies provide hypotheses on divergence times for major events in fungal evolution (e.g., the time for the origin of Fungi, Basidiomycota or Ascomycota), but not on the details of the radiation within a major group [26]–[27], [32], [46]–[47]. Most of these studies are also not focused on Fungi, but have a wider scope, including plants and animals, and the taxon sampling may not be optimal for calculating the origin and divergence times of lineages within Ascomycota or Basidiomycota [25], [48], [49]. One exception is the study of Gueidan et al. [50] in which several groups of Ascomycota, including lichen–forming fungi, were included with a broad taxon sampling. Gueidan et al. [50] still highlighted the need to improve the gene and taxon sampling, to achieve more accurate date estimates for fungal groups.

Several fossils related to extant mazaediate genera have been found in amber, but these have not been used for calibrating phylogenetic trees. We have recently produced a phylogeny of the Ascomycota with the aim of placing mazaedia–producing groups with previously unknown relationships [51]. This phylogeny contains representatives of several groups in which amber–preserved fossils occur, which can now be utilized for dating. Here, we want to take advantage of this recent phylogeny and use it to date the main diversification events within Ascomycota. To achieve this, we include all the dateable mazaediate fossils known, together with other Ascomycota fossils, to investigate the origin and divergence times of a number of major groups within Ascomycota, including lichenized fungi. We include a total of 6 fossils attributable to extant groups of Ascomycota. To account for the paleontological uncertainty, we also investigate the impact of various calibration scenarios on age estimates.

## Methods

### Taxon Sampling, Molecular Data and Phylogenetic Analysis

We included representatives of most lineages of Ascomycota, with a total of 118 species belonging to 10 classes of Pezizomycotina (Table S1) and three members of Saccharomycotina and Taphrinomycotina as the outgroup. Six loci were used for this analysis (nuLSU and nuSSU rDNA, the 5.8S nuclear rDNA, mtSSU rDNA, and the nuclear protein–coding genes RPB1, and MCM7) with a total of 4112 characters included. The alignments from [51] were utilized, with some additional sequences retrieved from GenBank (Table S1). All the alignments are available upon request. Substitution models for each partition were selected using the Akaike Information Criterion (AIC) implemented in jMODELTEST [52]. The GTR model [53] with an estimated proportion of invariable sites and with gamma-distributed rates among sites was selected for each of the six loci.

Phylogenetic relationships between taxa were estimated by maximum-likelihood analysis with the software RAxML VI–HPC [54]–[55] on the CIPRES Science Gateway v. 3.1 [56], using a GTR+I+G model of molecular evolution for six partitions (nuSSU, nuLSU, 5.8S, mtSSU, RPB1 and MCM7), rate heterogeneity with unlinked parameters and 1000 ML bootstrap replicates.

**Figure 1:**
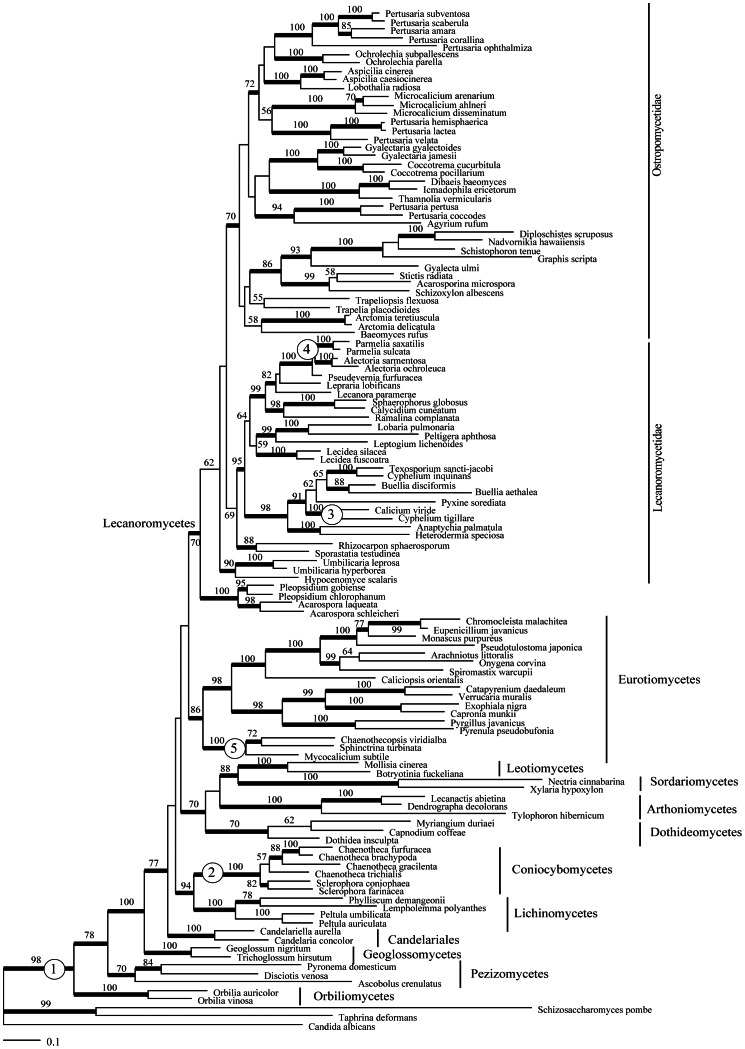
Best tree from the maximum-likelihood analysis. Numbers in circles indicate the nodes used for fossil calibration: (1) *Paleopyrenomycites devonicus*; (2) *Chaenotheca* sp.; (3) *Calicium* sp.; (4) *Alectoria succinica* or *Parmelia ambra*; and (5) *Chaenothecopsis* sp. Numbers above branches correspond to bootstrap support values and thicker branches show bootstrap support >70%.

We performed the RAxML analysis with 6 and with 10 partitions (i.e. dividing the three codon positions for each of the two protein coding genes), but since the topologies were identical, we only present and discuss the RAxML tree obtained with 6 partitions. The best tree with bootstrap support values is shown in Fig. 1.

### Molecular Clock Analysis

We implemented a Bayesian Markov chain Monte Carlo algorithm for estimating divergence times using data from multiple gene loci and accommodating multiple fossil calibration nodes. These analyses were performed using the BEAST v1.6.2 software package [23]. The tree topology and divergence times were estimated simultaneously. To provide an empirical test for the impact of various approaches to calibration, we investigated four different scenarios using several combinations of fossil constraints (see below, Table 1 and Fig. 1). In all cases, we partitioned the data by gene, with unlinked partitions with the GTR+I+G substitution model for each partition. We also explored one of the BEAST scenarios with 10 partitions, and the divergence times estimated were very similar to the same scenario with 6 partitions, so we used the latter partitioning scheme for all of the analyses. We used the uncorrelated lognormal relaxed clock model, which allows rates of molecular evolution to be uncorrelated across the tree. We implemented a birth–death tree prior. BEAST analyses were run for 50 million generations, logging parameters and trees every 1000 generations. Convergence, mixing and effective sample sizes (ESS) of parameters were checked using Tracer v1.5.0 [57]. A burn–in of 500 trees was removed from each analysis. The remaining trees were used to generate a maximum clade credibility tree with TreeAnnotator v1.6.2 [23]. The performance for each scenario was compared through a Bayes Factors analysis [58], carried out in Tracer v1.5.0, using the harmonic mean as an approximation of the marginal likelihood of each scenario, with 1000 bootstrap replicates to measure the error in the estimate (Table 2).

**Table 1:** Different scenarios investigated, fossils used, ages for each fossil and distributions associated with the selected fossils.

	Reference	Age (My)	Scenario 1	Scenario 2	Scenario 3	Scenario 4
*Alectoria succinica*	[40]	35–40	–	–	Exp (35, 33*)	Exp (35, 33*)
*Calicium* sp.	[44]	35–55	Exp (35, 33*)	Exp (35, 33*)	Exp (35, 33*)	Exp (35, 33*)
*Chaenotheca* sp.	[44]	35–55	Exp (35, 98.9^†^)	Exp (35, 98.9^†^)	Exp (35, 98.9^†^)	Exp (35, 98.9^†^)
*Chaenothecopsis* sp.	[39]	35–47	–	Exp (35, 98.9^†^)	–	Exp (35, 98.9^†^)
*Paleopyrenomycites devonicus*	[33]	400	Exp (400, 67.8^‡^)	Exp (400, 67.8^‡^)	Exp (400, 67.8^‡^)	Exp (400, 67.8^‡^)
*Parmelia ambra*	[42]	15–45	Exp (15, 39.4*)	Exp (15, 39.4*)	–	–

Exponential distribution = Exp (offset, mean). The means were selected so that 97.5% of the prior probability for each fossil date would fall below the age of the major group the fossil belongs to, i.e. 400 Mya which is the age for the †Pezizomycotina crown and 650 Mya for the ‡ Ascomycota base, both according to [32] and 160 Mya which is the age of the * Lecanorales based on [59].

**Table 2:** Natural logarithm (x2) of Bayes Factors obtained in Tracer for the 4 molecular clock Scenarios.

	Marginal likelihood	S.E.	Scenario 1	Scenario 2	Scenario 3	Scenario 4
Scenario 1	−97269,904	+/−0,237	–	−1,69	−1,59	−1,12
Scenario 2	−97269,059	+/−0,217	1,69	–	0,1	0,57
Scenario 3	−97269,109	+/−0,246	1,59	−0,1	–	0,47
Scenario 4	−97269,344	+/−0,216	1,12	−0,57	−0,47	–

Marginal likelihood estimated as the ln harmonic mean likelihoods of the data. S.E.: Standard error of the marginal likelihood.

### Fossil Calibration

For this study we selected the six fossils we considered most reliable in age and identification, including lichenized and non–lichenized Ascomycota (Table 1, Fig. 1). To incorporate uncertainty surrounding fossil calibrations, we specified prior probability distributions with a hard minimum bound, i.e. the estimated divergence date cannot be younger than the earliest known fossil. The correct use of fossils to calibrate phylogenetic trees is much debated (e.g. a summary in [18]). Several parametric distributions are available as priors on nodal ages in a Bayesian phylogenetic framework [16]. In the present study we carried out several analyses, hereafter called “scenarios”, including different combinations of fossils (Table 1). All our scenarios share *Paleopyrenomycites devonicus* from the lower Devonian (400 Mya) as the oldest calibration point. Scenario 1 included the calibration points *Calicium sp.*, *Chaenotheca sp.* and *Parmelia ambra* with exponential distributions associated with their priors. In Scenario 2 we added *Chaenothecopsis* as another calibration point to the previous scenario. Scenario 3 includes *Alectoria succinica*, *Calicium sp.* and *Chaenotheca sp.* with exponential distributions. In Scenario 4 we added the *Chaenothecopsis* calibration point to Scenario 3.

*Paleopyrenomycites devonicus* was placed at the base of the stem group, corresponding to the Pezizomycotina–Saccharomycotina divergence (the “Pezizomycotina origin”, constraint 1 in Fig. 1). *Chaenotheca sp.* [44] was placed at the base of the family Coniocybaceae (constraint 2 in Fig. 1) and *Calicium sp.* [44] was used to calibrate the *Calicium*–*Cyphelium* clade (constraint 3 in Fig. 1). Amo de Paz et al. [59] used *Alectoria succinica* [40] as a calibration point either for the *Alectoria* clade or the whole alectorioid clade. As there is an uncertainty in this assignment, we decided to use it to calibrate the stem group (i.e. the *Parmelia*–*Alectoria* clade, constraint 4 in Fig. 1) to be more conservative. *Parmelia ambra* [42] was used by Amo de Paz et al. [59] to calibrate the crown of *Parmelia* sensu stricto. Poinar et al. [42] pointed out that *Parmelia ambra* appears very similar to extant members of *Parmelia* and it is tempting to speculate on its resemblance to *P. saxatilis* and similar species. However, the small specimen studied lacks necessary characters to ensure that it belongs to *Parmelia*. Taking this uncertainty into account, we took a conservative approach and used this fossil to calibrate the stem of this group (i.e. the *Parmelia–Alectoria* clade, constraint 4 in Fig. 1), which is beyond doubt. Given that we cannot use two different fossils to calibrate the same node, we use both mutually exclusive scenarios, with either *Parmelia* or *Alectoria* (Table 1, Scenarios 1, 2, 3, 4 and constraint 4 in Fig. 1). We also incorporated *Chaenothecopsis* (Table 1, Scenarios 2 and 4), from Eocene Baltic and Oligocene Bitterfeld ambers [39], to calibrate the Mycocaliciales clade (constraint 5, Fig. 1).

For nodes with less precise ages (e.g. known from one or a small number of fossils) Brown & Sorhannus [60] suggested using two distinct calibration procedures, exponential and lognormal. The lognormal distribution is considered appropriate for use as a prior on calibrated nodes because it places the highest probability on ages somewhat older than the fossil [16]. However, the exponential distribution (with the highest probability similar to fossil age) can be a good alternative to a lognormal when there is insufficient paleontological information, as this distribution requires one fewer parameter. Hence, we chose an exponential distribution to minimize the number of additional parameters estimated from the data (Table 1, scenarios 1, 2, 3 and 4).

## Results and Discussion

The best tree identified in the maximum-likelihood analysis (Fig. 1) is congruent with recent larger phylogenetic studies of the Ascomycota [51], [61]–[63]. The 11 Pezizomycotina classes included in the study were recovered as monophyletic. A discussion of the detailed relationships among euascomycete classes can be found in [51].

### Comparing Scenarios

**Figure 2:**
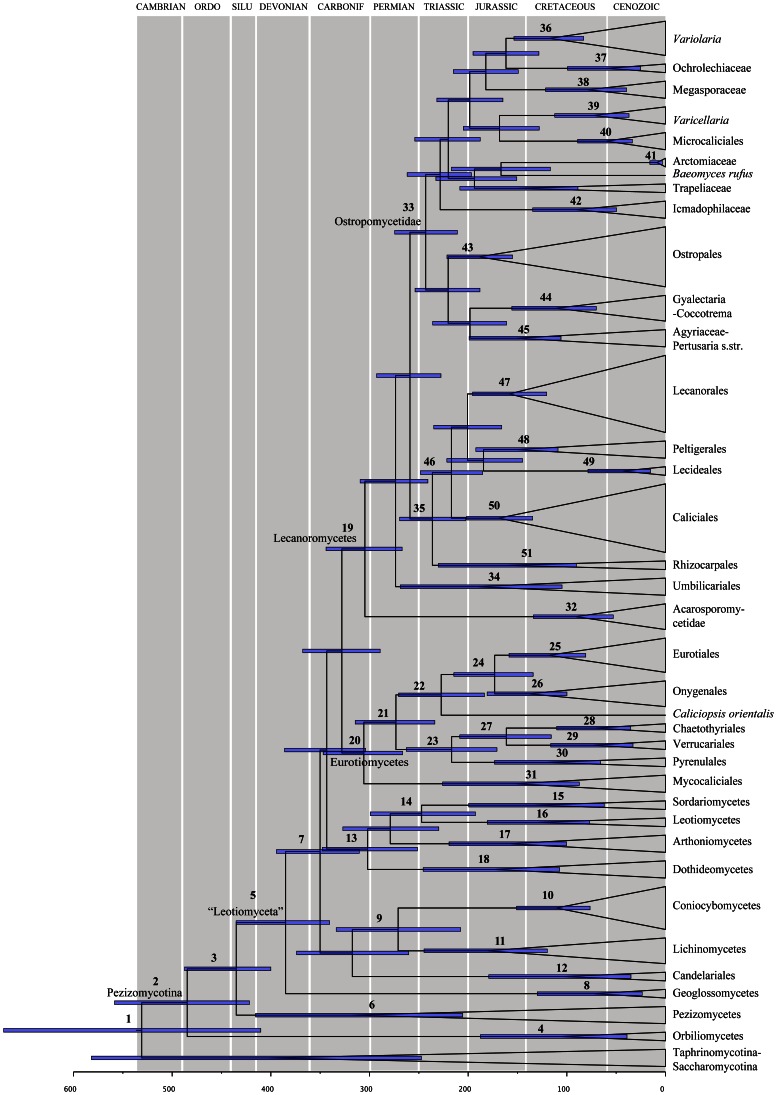
Maximum clade credibility chronogram for the major groups of Ascomycota. The chronogram is the result from the BEAST analysis of Scenario 4. Each node represents the mean divergence time estimate and bars show their associated 95% credibility interval. Numbers corresponding to dated groups shown in Table 3 are written above the nodes.

**Figure 3:**
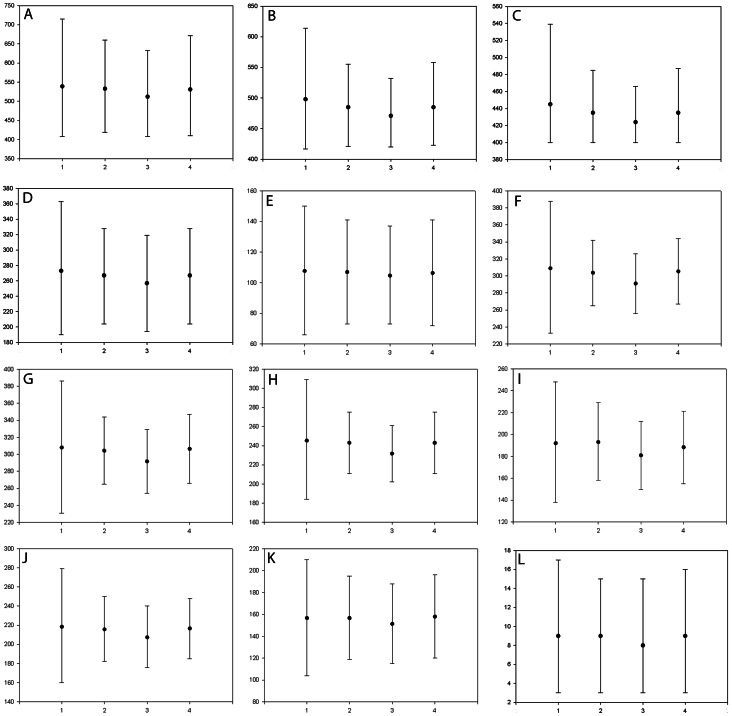
Comparison of divergence ages in several Ascomycota clades. The mean divergence time estimates and their associated 95% credibility intervals (y–axis) for selected nodes within Ascomycota for the 4 scenarios studied (x–axis) are represented. A: node 1, Ascomycota crown; B: node 2, Pezizomycotina crown; C: node 3, Pezizomycetes–“Leotiomyceta”; D: node 9, Lichinomycetes–Coniocybomycetes; E: node 10, Coniocybomycetes crown; F: node 19, Lecanoromycetes crown; G: node 20, Eurotiomycetes crown; H: node 33, Ostropomycetidae; I: node 43, Ostropales; J: node 46, Lecanoromycetidae crown; K: node 47, Lecanorales crown; L: node 41, Arctomiaceae.

To assess the fit of the different calibration scenarios we compared the Bayes Factors of the –log likelihood between each scenario (Table 2, expressed as twice the natural logarithm of the Bayes factor). Age estimates with means and 95% confidence intervals for some of the major Ascomycota splits from the 4 scenarios are summarized in Table 3 and the chronogram from Scenario 4 is shown in Fig. 2. The Bayes factors analysis does not favour any particular scenario according to the criteria by Kass & Raftery [58] (Table 2). The four scenarios yield date estimates with overlapping credibility intervals, but the means and range of the age estimates vary with the different fossil constraints used (Fig. 3, Table 3). Mean estimates are younger and the credibility intervals are narrower in Scenario 3 than in Scenario 1 (with *Alectoria* or *Parmelia*, respectively, as constraints) (Fig. 3, Table 3). Scenario 2 differs very little from Scenario 4 (*Parmelia* or *Alectoria*, plus *Chaenothecopsis*, respectively, as constraints) (Fig. 3, Table 3). Scenario 2 has narrower credibility intervals than Scenario 1, both at basal nodes and nodes towards the tips, but Scenario 4 has wider credibility intervals (especially in some basal nodes) and older means than in scenario 3. We interpret this as meaning that the inclusion of a more accurately dated fossil (*Alectoria*) affects the dating estimates differently depending on the number of fossils included, and that the effect is reduced when an additional fossil (*Chaenothecopsis*) is included. The effect also depends on the node being considered, as nodes closer to the tips are less affected than nodes further down the tree. We can conclude as a general rule that it is important to select as accurately dated fossils, and to include as many fossils, as possible.

**Table 3:** Mean and range (95% credibility intervals) divergence time estimations (Mya) among the major Ascomycota lineages for the 4 molecular clock scenarios studied.

	Scenarios	Scenario 1		Scenario 2		Scenario 3		Scenario 4	
Node label	Clade name	95% CI	Mean	95% CI	Mean	95% CI	Mean	95% CI	Mean
**1**	Ascomycota crown	408–715	539	419–660	533	408–633	512	410–671	531
**2**	Pezizomycotina crown	417–614	498	421–555	485	420–532	471	422–558	485
**3**	Pezizomycetes–Leotiomyceta	400–539	445	400–485	435	400–466	424	400–487	435
**4**	Orbiliomycetes crown	32–201	107	41–197	110	39–191	105	39–188	106
**5**	Leotiomyceta crown	306–490	388	338–434	383	325–413	370	338–434	383
**6**	Pezizomycetes crown	192–448	322	203–425	322	213–407	317	203–425	322
**7**		274–444	353	308–385	348	298–373	335	310–394	350
**8**	Geoglossomycetes crown	27–145	77	26–127	70	29–142	77	26–127	70
**9**	Lichinomycetes–Coniocybomycetes	190–363	273	204–328	267	194–319	257	204–328	267
**10**	Coniocybomycetes crown	66–150	107	73–141	107	73–137	104	72–141	106
**11**	Lichinomycetes crown	107–260	181	114–238	175	112–228	170	114–238	175
**12**	Candelariales crown	30–196	105	36–178	101	40–164	98	36–178	101
**13**	Inoperculate Fungi+Arthoniomycetes	224–386	302	255–346	300	243–331	288	251–348	302
**14**	Sordariomycetes–Leotiomycetes split	166–320	245	197–296	245	190–285	236	193–299	247
**15**	Sordariomycetes crown	64–194	127	81–189	135	71–170	121	77–181	130
**16**	Leotiomycetes crown	61–209	134	67–191	129	69–197	130	62–199	133
**17**	Arthoniomycetes crown	88–232	158	96–228	161	95–214	153	100–219	158
**18**	Dothideomycetes crown	93–252	169	105–245	173	103–226	164	107–245	174
**19**	Lecanoromycetes crown	233–388	306	265–342	304	256–326	291	267–344	305
**20**	Eurotiomycetes crown	231–386	307	265–344	304	254–329	292	266–347	306
**21**		204–354	276	233–314	273	224–305	263	234–314	273
**22**		165–303	230	186–272	229	179–261	221	184–270	227
**23**		155–292	222	173–262	217	167–253	210	171–263	217
**24**		121–233	174	137–215	175	129–207	168	134–214	173
**25**	Eurotiales	73–169	119	84–163	122	80–154	117	81–159	118
**26**	Onygenales	89–193	139	102–183	140	95–176	134	100–181	139
**27**		107–228	166	120–208	163	114–204	157	116–208	161
**28**	Chaetothyriales	33–121	74	38–114	75	32–106	69	35–110	72
**29**	Verrucariales	35–124	76	37–112	72	36–107	70	33–116	74
**30**	Pyrenulales	58–185	120	65–172	119	63–167	115	66–173	120
**31**	Mycocaliciales	84–239	157	82–232	153	79–218	146	87–226	152
**32**	Acarosporomycetidae	49–146	95	55–132	91	52–134	90	53–134	91
**33**	Ostropomycetidae	184–309	243	211–275	243	202–261	232	211–275	243
**34**	Umbilicariales crown	94–280	194	102–266	192	106–253	182	105–269	191
**35**	Lecanoromycetidae* crown	175–302	235	202–269	234	193–257	225	203–269	236
**36**	Variolaria	75–167	121	82–155	118	82–150	115	83–154	118
**37**	Ochrolechiaceae	22–99	59	22–99	58	25–95	58	25–99	59
**38**	Megasporaceae	37–132	80	42–132	80	41–122	79	39–121	80
**39**	Varicellaria	36–122	76	35–115	74	33–113	71	37–112	73
**40**	Microcaliciales	31–98	63	34–92	61	32–88	59	34–89	60
**41**	Arctomiaceae	3–17	9	3–15	9	3–15	8	3–16	9
**42**	Icmadophilaceae	45–145	90	51–136	91	47–125	85	50–135	90
**43**	Ostropales	138–248	190	158–229	192	150–212	181	155–221	189
**44**	Coccotremataceae	66–163	112	72–159	113	69–154	109	70–156	111
**45**	Agyriaceae–Pertusaria s.str.	91–205	147	99–196	148	95–189	141	106–199	151
**46**	Lecanoromycetidae** crown	160–279	216	182–250	215	176–240	206	185–248	217
**47**	Lecanorales crown	104–210	156	119–195	156	115–188	151	120–196	158
**48**	Peltigerales crown	95–201	146	104–193	149	101–189	142	109–192	150
**49**	Lecideales crown	16–79	44	16–73	42	15–71	42	15–79	44
**50**	Caliciales crown	118–222	168	132–199	166	128–193	161	135–202	169
**51**	Rhizocarpales crown	66–239	156	84–224	162	87–217	156	90–230	161

Node labels correspond to nodes in Fig. 2.

* Excluding Umbilicariales;

** Excluding Umbilicariales and Rhizocarpales.

### Divergence Times of Major Ascomycota Groups

The maximum clade credibility trees obtained for all the BEAST analyses are topologically identical to the best tree obtained with maximum likelihood. As scenario 4 includes most fossils, and the most accurate dated fossil (*Alectoria*), we will base our discussion below on the dates obtained from scenario 4 (Fig. 2; chronogram).

For all scenarios, the first divergence in Ascomycota (the split between [Saccharomycotina+Taphrinomycotina] and Pezizomycotina) took place in the Early Cambrian (node 1; 531 Mya; 410–671 Mya credibility interval [CI]), which corresponds to the origin of the Pezizomycotina (the euascomycetes, or the Ascomycota crown). In previous studies, various dates have been proposed for this divergence, ranging from ca. 325 [24] to 1316 Mya [27]. Lücking et al. [32] estimated that this split happened ca. 400–520 Mya (Pezizomycotina stem base), and Gueidan et al. [50], using relaxed clock methods, estimated this date to be 538 Mya, which are very close to the estimates obtained here. These results place the origin of the Pezizomycotina in the Cambrian, which is earlier than the origin and diversification of the land plants. As the first groups to diverge, Taphrinomycotina and Saccharomycotina, include both saprotrophs and parasites, this dating is consistent with the hypothesis that the ancestors of the Pezizomycotina would have been saprotrophs on algae or invertebrates. But whether the origin of the ancestors of Ascomycota and Pezizomycotina was marine or terrestrial is an open question. Some propose a terrestrial origin of the Ascomycota (e.g. [64]), but this hypothesis has never been tested with adequate methodology to confidently resolve the ancestry for Ascomycota. Marine fungi have been reported from oceanic areas in different parts of the world, living as saprophytes and parasites, contributing to the decay of algae or other plant remains and the infection of marine plants and animals. As the dating obtained here for the Pezizomycotina is earlier than the appearance of land plants, this suggests the possibility of a marine origin for the group. Further work is needed to properly test the possible marine–terrestrial transition within the early evolution of the Ascomycota.

The earliest splits (Fig. 2) within Pezizomycotina took place in the Ordovician, resulting in the Orbiliomycetes and the Pezizomycotina crown group (node 2; 485 Mya, 422–558 CI); in the Silurian (node 3; 435 Mya, 400–487 CI), resulting in the Pezizomycetes and the “Leotiomyceta”; and in the Upper Devonian (node 5, 383 Mya; 338–434 Mya CI) giving rise to the Geoglossomycetes. A recent date estimation of the origin of the Pezizomycetes [50] was very similar to ours (441 Mya, 386–498 Mya CI), but the divergence between Orbiliomycetes and the Pezizomycotina crown group was slightly younger (455 Mya, 396–516 Mya).

Character state reconstruction supports a saprobic nutritional mode for ancestors of “Leotiomyceta” and Pezizomycetes [62]. At the time we estimate that they appeared (i.e. Silurian) there is direct fossil evidence of vascular plants and terrestrial arthropods [65] with which these fungi could have established biotic interactions, thus also supporting that both these groups had saprophytic ancestors.

The lineage including, among others, all lichenized lineages in the tree (node 7) is dated to 350 Mya, 310–394 CI, in the early Carboniferous. This lineage also includes non-lichenized fungi and the ancestor is considered to have been non–lichenized [62], [66]. The stem leading to most members of inoperculate fungi (i.e. Dothideomycetes, Leotiomycetes and Sordariomycetes), and the lichenized class Arthoniomycetes, dates from the Upper Carboniferous (node 13, 302 Mya). The most recent common ancestor for the lichen classes Lichinomycetes and Coniocybomycetes dates from the Permian (node 9; 267 Mya, 204–328 CI). The diversification of Eurotiomycetes and Lecanoromycetes took place in the Upper Carboniferous (nodes 20 and 19, 306 and 305 Mya respectively). The Lecanoromycetes include most lichenized Ascomycota, and the ancestor has been reconstructed as lichenized [66], suggesting that the main lineages of lichen–forming Ascomycota originated in the Upper Carboniferous. During this period, the uplift of the continents caused a transition to a more terrestrial environment, and a trend towards aridity. The lycopods underwent a major extinction event and ferns became more important with an increase in the number of trees. By the end of the Carboniferous, all five of the major extant fern lineages were present [67], and gymnosperms had appeared [68], which resulted in a wide range of substrates to colonize and thus could explain the rapid diversification of the Lecanoromycetes at this time.

Although node 35 is not fully supported (69% bootstrap support in the likelihood analysis, Fig. 1), lineages leading to Lecanoromycetidae (excluding the Umbilicariales but including Rhizocarpales) originated during the Triassic, after the P–T mass extinction (node 35, 236 Mya), as well as the diversification of Ostropomycetidae (node 33, 243 Mya) and the diversification of Lecanoromycetidae (excluding Rhizocarpales, node 46, 217 Mya).

Within the Eurotiomycetes, all orders diversified during the Cretaceous (nodes 24, 25, 27, 28, 29 and 30). The main orders within the Lecanoromycetidae (47, 48 and 50 dated from 158, 150 and 169 Mya respectively) diversified in the Jurassic, except the Lecideales (node 49, 42 mya) which diversified in the Eocene. The relationships within Ostropomycetidae are not fully resolved, as in other studies [69]–[71], and divergence times have been obtained mainly at the family level. Our results indicate that most of the families diverged already in the Cretaceous–Paleocene (nodes 36 to 40), except the Arctomiaceae which diverged in the Miocene (node 41, 9 Mya).

Although all major lichenized lineages had their origins in the Upper Carboniferous (Table and Fig. 3), it was successive radiations in the Jurassic and Cretaceous that generated the diversity in the main modern groups. Recent estimates suggest that the origin of angiosperms was either in the lower Jurassic to Lower Cretaceous [72]–[74], or in the Triassic–early Jurassic [75]. This could explain the main diversification events within Lecanoromycetidae and Ostropomycetidae, in which many taxa grow as epiphytes. The high levels of diversity in lichenized groups in the Jurassic and Cretaceous could thus be potentially explained by the many new environments dominated by the new diversity of angiosperms, which could be inhabited by these lichens.

In our analysis, Acarosporomycetideae and Candelariales are two of the oldest groups of lichenized fungi, but their crown diversification dates are among the most recent (nodes 11 and 31, Upper Cretaceous). Whether these two clades have experienced a recent speciation or suffered a high number of extinctions is unknown and further studies are necessary to answer this question.

### Conclusions

Despite the increasing interest in dating the origin and diversification of different groups in Fungi [59], [76]–[79], we still lack detailed information to provide hypotheses on the evolutionary history of Ascomycota. This includes the lichenized euascomycetes, a clade that is key to understanding the evolution of symbiosis in Fungi. In this study we provide three important observations that can further our knowledge of the evolutionary rate and dates in Ascomycota. First, our analysis suggests an ancient origin of Pezizomycotina, ca 530 Mya, in the Cambrian period. Second, the main lichenized Ascomycota lineages appeared at least as early as the Carboniferous, with successive radiations in the Jurassic and Cretaceous that generated the diversity in the main modern groups. Third, we provide estimates for the origin and diversification dates for many clades in the Pezizomycotina, which previously had not been dated, including the current major classes and orders, as well as some families of both lichenized and non–lichenized groups. Such dates, even if tentative, set a promising foundation for future hypotheses on the evolution of this fascinating group of Fungi.

## Supporting Information

Table S1 **Specimens used in this study with GenBank accession numbers.**
(XLS)
